# Newborn Screening in Japan—2021

**DOI:** 10.3390/ijns8010003

**Published:** 2022-01-04

**Authors:** Toshihiro Tajima

**Affiliations:** Jichi Children’s Medical Center Tochigi, Jichi Medical University, Shimotsuke-shi 329-0498, Japan; t-tajima@jichi.ac.jp

Japan’s Newborn Mass Screening (NBS) was started in 1977 for amino acid metabolism disorders (phenylketonuria (PKU), homocystinuria, maple syrup urine, histidineemia (discontinued in 1993)) and galactosemia at the national level as a national project. Subsequently, congenital hypothyroidism was added in 1979, congenital adrenal hyperplasia was added in 1989, and screening was being conducted for six diseases.

From 2014, a tandem mass analyzer (tandem mass) was introduced nationwide in place of the conventional Guthrie test, and in addition to the conventional amino acid metabolism disorders, urea cycle disorders, organic acid metabolism disorders and fatty acid metabolism disorders have joined the target diseases. Screening is currently being conducted for 20 diseases. The acceptance rate of mass screening in Japan is 100%, and the world’s top-level screening, such as quality control system and inspection system, is being carried out.

The Japanese Society for Neonatal Screening was established in 1973 and is now a subcommittee of the Japan Pediatric Society. Members are composed of clinicians (pediatrics, obstetrics and gynecology, internal medicine, etc.), laboratory technicians, basic medical researchers, public health/epidemiological researchers, and administrative personnel.

At this time, the past, present and future of mass screening in Japan is summarized in the Topical Collection of the *International Journal of Neonatal Screening*. I would like to thank everyone involved.

This Topical Collection includes the following topics that are important in newborn screening:

- Long-term prognosis of congenital metabolic diseases found by newborn screening
- Screening issues for congenital hypothyroidism and congenital adrenal hyperplasia
- Challenges of tandem mass screening and new knowledge in Japan
- Screening for spinal muscular atrophy (SMA)

Examination of the long-term prognosis of the diseases found in NBS is an important point. Yamada et al. have investigated the prognosis of adult patients with homocystinuria (HCU) [1] and phenylketonuria (PKU) [2]. HCU revealed that cases found by NBS had a generally good neurological prognosis and general social life. However, it was suggested that Marfanoid and psychiatric symptoms may worsen with age even with treatment. In PKU, pre-NBS patients had some neurological complications, but many patients found by NBS lived a normal social life without neurological problems. However, even patients found with NBS had neuropsychiatric complications in those who discontinued treatment.

It is known that there are many carriers of the PAH gene c.158G> A (p.R53H) in East Asia. Hyperpheylalaninemia (HPA) was also found in the NBS of PKU, but its follow-up policy is not clear in some parts. Odagiri et al. [3] analyzed the genotype and phenotype of Japanese PKU and HPA patients in a large number of cases. As a result, HPA carrying a variant of p.R53H was untreated, and its Phe level was below 360 μmol/L. Therefore, they propose a follow-up method of HPA.

Congenital hypothyroidism (CH) is the most common disease found by NBS. Minamitani [4] discusses changes in the TSH cut-off of CH screening in Japan, an overview of guidelines for CH diagnosis and treatment, screening for low-birth-weight infants and an increase in the frequency of CH in Japan, as in the rest of the world. Furthermore, Nagasaki et al. [5] follow up with patients with CH found in NBS up to the age of 15 and re-evaluate during that course to examine the frequency of permanent CH and transient CH. As a result, it was shown that the frequency of permanent CH is 1 in 2500–3000, which is almost the same as in other countries. Regarding congenital adrenal hyperplasia (CAH), Tsuji-Hosokawa et al. [6] have reported the data of 30 years in the Tokyo Metropolitan area. One of the problems with CAH’s NBS is that it has a high false positive rate. Simultaneous measurement of 17-OHP and other steroids has been successful in reducing the false positive rate.

On the topic of tandem screening, Shigematsu et al. [7] have reported in detail the development of a new secondary test method using liquid chromatography-tandem mass spectrometry for the purpose of reducing the false positive rate. Tajima et al. [8] have described the current state of NBS in Japan for Propionic acidemia. The PCCB gene c.1304T> C (p.Y435C) variant is particularly frequently found in Japanese people, so Propionic acidemia is found in 1 in 45,000 people. The cases harboring this variant are considered to be mild, but the treatment and follow-up policy has not yet been determined.

Kagawa et al. [9] have reported cases of cobalamin disorders and methylenetetrahydrofolate reductase deficiency that cannot be detected by the current tandem mass screening. They are investigating improvements in tandem mass screening to detect these cases.

Nucleic acid drug therapy and gene replacement therapy have been developed for SMA, and NBS has been introduced in other countries. On the other hand, although the introduction to NBS is delayed in Japan, Kimizu [10] et al. have reported the frequency of SMA in Japan and the pilot study of NBS and discussed how to introduce NBS for SMA in Japan.

We would like to thank the authors for providing the cutting-edge article series including NBS achievements, problems, solutions, and new screenings that will be developed in the future in Japan. We would also like to thank the reviewers for carefully reviewing the submitted papers and increasing their scientific value.

We will continue to make efforts so that the Japanese Society for Neonatal Screening can contribute to newborn screening. We look forward to overcoming the COVID-19 pandemic and meeting you at the Scientific Meeting of the International Society for Neonatal Screening.
